# Assessing Dietary Outcomes in Intervention Studies: Pitfalls, Strategies, and Research Needs

**DOI:** 10.3390/nu10081001

**Published:** 2018-07-31

**Authors:** Sharon I. Kirkpatrick, Clare E. Collins, Ruth H. Keogh, Susan M. Krebs-Smith, Marian L. Neuhouser, Angela Wallace

**Affiliations:** 1 School of Public Health and Health Systems, University of Waterloo, 200 University Avenue West, Waterloo, ON N2L 3G1, Canada; 2 Priority Research Centre for Physical Activity and Nutrition, University of Newcastle, University Drive, Callaghan, NSW 2308, Australia; clare.collins@newcastle.edu.au; 3 Department of Medical Statistics, London School of Hygiene and Tropical Medicine, Keppel Street, London WC1E 7HT, UK; ruth.keogh@lshtm.ac.uk; 4 Division of Cancer Control and Population Sciences, National Cancer Institute, 9609 Medical Center Drive, Room 4E142 Bethesda, MD 20892-9763, USA; krebssms@mail.nih.gov; 5 Division of Public Health Sciences, Fred Hutchinson Cancer Research Center, 1100 Fairview Ave North, M4B402, Seattle, WA 98109, USA; mneuhous@fredhutch.org; 6 Family Relations and Applied Nutrition, University of Guelph, 50 Stone Road East, Guelph, ON N1G 2W1, Canada; angelaw@uoguelph.ca

**Keywords:** interventions, dietary assessment, dietary outcomes, measurement error, bias, differential reporting

## Abstract

To inform strategies to improve the dietary intakes of populations, robust evaluations of interventions are required. This paper is drawn from a workshop held at the International Society of Behavioral Nutrition and Physical Activity 2017 Annual Meeting, and highlights considerations and research priorities relevant to measuring dietary outcomes within intervention studies. Self-reported dietary data are typically relied upon in such studies, and it is recognized that these data are affected by random and systematic error. Additionally, differential error between intervention and comparison groups or pre- and post-intervention can be elicited by the intervention itself, for example, by creating greater awareness of eating or drinking occasions or the desire to appear compliant. Differential reporting can render the results of trials incorrect or inconclusive by leading to biased estimates and reduced statistical power. The development of strategies to address intervention-related biases requires developing a better understanding of the situations and population groups in which interventions are likely to elicit differential reporting and the extent of the bias. Also needed are efforts to expand the feasibility and applications of biomarkers to address intervention-related biases. In the meantime, researchers are encouraged to consider the potential for differential biases in dietary reporting in a given study, to choose tools carefully and take steps to minimize and/or measure factors such as social desirability biases that might contribute to differential reporting, and to consider the implications of differential reporting for study results.

## 1. Introduction

Governments around the world are considering an array of strategies to shift the dietary intakes of populations to improve health and reduce chronic disease risk [1]. To develop a stronger evidence base to inform such efforts, robust evaluations of the impacts of interventions on dietary outcomes are required. This paper, drawn from discussions at a workshop held at the International Society of Behavioral Nutrition and Physical Activity 2017 Annual Meeting, highlights considerations and research priorities relevant to measuring dietary outcomes within intervention studies.

## 2. Pitfalls in Assessing Dietary Outcomes in Intervention Research

There is a reliance on self-report dietary assessment instruments in most population-based nutrition research, since objective measures are limited, burdensome, and/or expensive. It is well-recognized that self-reported dietary data are affected by random and systematic error [2]. In the fields of surveillance and epidemiology, significant attention has been given to these types of error and their impacts, leading to efforts to improve measurement tools to reduce error and to develop statistical techniques to mitigate the impact of error on study results [3,4].

Within intervention research, a unique source of measurement error for which mitigation strategies are currently lacking stems from potential differences in dietary reporting as a result of exposure to the intervention itself [5,6]. For example, those participating in nutrition education, counselling, or other initiatives that aim to promote healthy eating may report their diets differently post-intervention or compared to those in control or comparison groups. The Women’s Health Initiative Nutritional Biomarkers Study, which compared self-reported energy intake to estimates from doubly labeled water (DLW), found that women in the intervention arm (who participated in group and individual nutrition intervention sessions and self-monitored their fat, fruit, and vegetable intakes [7]) underreported total energy intake by about 5% more than those in the comparison arm [5]. On the other hand, in the Women’s Healthy Eating and Living Study, in which those in the intervention arm were “counseled to adopt a micronutrient and phytochemical-rich diet”, the accuracy of carotenoid intake estimates (relative to plasma levels) from self-report data improved among those in the intervention arm [6]. 

These studies suggest that participation in an intervention can influence how individuals report their dietary intake. This differential reporting may come about because of improved accuracy of self-reporting as a result of exposure to the intervention (for example, through greater awareness of eating or drinking occasions), the desire to appear compliant [6], or other facets of social desirability. To the extent that foods and beverages perceived as less healthy are misreported to a greater degree than those perceived as healthy, the displacement of less-healthy items in response to the intervention (e.g., replacement of saturated fats with mono- or poly-unsaturated fats or of soda and juice with milk) could contribute to more accurate post-intervention reporting of intake. Differential reporting is problematic because it can render the results of trials distorted or inconclusive [6] by leading to biased estimates of the intervention effect and reduced statistical power.

## 3. Moving Forward

Statistical approaches to correct for the impact of differential measurement error are currently limited and rely on the availability of unbiased measures of intake. For example, combining self-report data with data from recovery biomarkers (which provide unbiased estimates of absolute intake) may be helpful, as demonstrated by Keogh et al. [8]. To expand our toolkit for understanding and addressing differential error, there is a need for studies that entail the collection of self-report data and unbiased markers of intake (e.g., recovery biomarkers or real-time unobtrusive observation to document true intake) within the context of intervention studies aimed at changing diet. Such data would be invaluable for characterizing the situations and population groups in which interventions are likely to elicit differential reporting and the extent of the differences, as well as to shed light on the effectiveness of potential strategies to reduce or mitigate differential error, such as measuring and accounting for social desirability [9]. The identification of a broader array of recovery biomarkers is also needed, in addition to strategies to make their collection feasible and cost-effective in a wider range of studies. Efforts to examine how individual factors, such as age, sex, body mass index, and genetic and phenotypic factors, affect concentrations of non-recovery biomarkers could inform their broader use to understand intervention effects on dietary outcomes. Such efforts may benefit from international partnerships to leverage existing data sources as well as relevant statistical and analytic expertise to shed light on intervention-related biases.

Discussions throughout this workshop highlighted the need for practical strategies in the interim for researchers conducting studies in which the accurate measurement of dietary outcomes is fundamental. One important factor to consider at an early stage is the extent to which a given intervention is likely to elicit differential error. For example, interventions targeting features of the food environment (e.g., taxation or product placement) may be less likely to elicit differential error compared to those that target behavior change. If differential error is likely, this should be considered throughout the study, from design to interpretation.

In any intervention study, the dietary assessment tools used should provide the most accurate data possible for the dietary components of interest, and should demonstrate responsiveness to true changes in diet given realistic sample sizes and statistical power. Administration issues should also be considered. For example, unannounced versus pre-scheduled 24-h recalls can reduce alteration of diet, or reactivity, in response to reporting [6]. Another consideration is the provision of resources to help participants report as accurately as possible. For example, participants in studies using self-administered tools may benefit from quick-start guides or videos to help them navigate the interface. Resources available to assist researchers in making decisions about tools include the National Cancer Institute’s Dietary Assessment Primer (https://dietassessmentprimer.cancer.gov/), the Medical Research Council’s Diet and Physical Activity Toolkit (http://dapa-toolkit.mrc.ac.uk/), and the Australasian Child and Adolescent Obesity Research Network Dietary Intake Assessment Method Selection Guide (http://anzos.com/acaorn/food-and-nutrition/), although it should be noted that these resources are currently limited in their discussions of strategies to optimize the measurement of dietary outcomes.

In cases in which differential error is likely, the inclusion of objective measures (possibilities aside from biomarkers and real-time observation include data on food sales and/or purchasing in studies in which these may be relevant) can be used to corroborate findings based on self-reported dietary intake. It is also possible to measure social desirability [9] and account for this potential source of error in the analysis and interpretation of dietary data, but it must be borne in mind that there are other possible contributors to intervention-related biases. Furthermore, the tools used to assess social desirability must do so accurately and reliably.

When reporting the results of intervention studies, it is critical to consider the potential implications of differential measurement error in order to avoid erroneous interpretations. To this end, the tailoring of reporting checklists such as the Consolidated Standards of Reporting Trials (CONSORT) [10] to the context of nutrition research may be warranted. Additionally, the Strengthening the Reporting of Observational Studies in Epidemiology (STROBE) Statement-nutritional epidemiology (STROBE-nut) checklist [11], though tailored to epidemiology, offers guidance relevant to any study that relies upon the assessment of dietary intake. Relevant STROBE-nut guidelines include describing the main limitations of the assessment methods used and any implications for the interpretation of findings. To encourage transparency, such checklists and corresponding details within manuscripts should increasingly be viewed as necessary elements of submissions reporting on the dietary outcomes of intervention trials.
